# A human-ACE2 knock-in mouse model for SARS-CoV-2 infection recapitulates respiratory disorders but avoids neurological disease associated with the transgenic K18-hACE2 model

**DOI:** 10.1128/mbio.00720-25

**Published:** 2025-04-24

**Authors:** Anna Pons-Grífols, Ferran Tarrés-Freixas, Mònica Pérez, Eva Riveira-Muñoz, Dàlia Raïch-Regué, Daniel Perez-Zsolt, Jordana Muñoz-Basagoiti, Barbara Tondelli, Edwards Pradenas, Nuria Izquierdo-Useros, Sara Capdevila, Júlia Vergara-Alert, Victor Urrea, Jorge Carrillo, Ester Ballana, Stephen Forrow, Bonaventura Clotet, Joaquim Segalés, Benjamin Trinité, Julià Blanco

**Affiliations:** 1 IrsiCaixa, Can Ruti Campus, Badalona, Spain; 2 Unitat mixta d'investigació IRTA-UAB en Sanitat Animal, Centre de Recerca en Sanitat Animal (CReSA), Campus de la Universitat Autònoma de Barcelona (UAB), Bellaterra, Spain; 3 IRTA, Programa de Sanitat Animal, Centre de Recerca en Sanitat Animal (CReSA), Campus de la Universitat Autònoma de Barcelona (UAB), Bellaterra, Spain; 4 University of Vic-Central University of Catalonia (UVic-UCC) 16783, Vic, Spain; 5 Mouse Mutant Core Facility, Institute for Research in Biomedicine (IRB Barcelona), the Barcelona Institute of Science and Technology, Barcelona, Spain; 6 Germans Trias i Pujol Research Institute (IGTP), Badalona, Spain; 7 CIBER Infectious Diseases (CIBERINFEC), Carlos III Health Institute, Madrid, Spain; 8 Comparative Medicine and Bioimage Centre of Catalonia (CMCiB), Germans Trias i Pujol Research Institute (IGTP), Badalona, Spain; 9 Departament de Sanitat i Anatomia Animals, Facultat de Veterinària, Universitat Autònoma de Barcelona (UAB), Campus de la UAB, Bellaterra, Spain; University of Florida College of Public Health and Health Professions, Gainesville, Florida, USA

**Keywords:** animal model, lung inflammation, lung damage, neuroinvasion, post-COVID condition

## Abstract

Animal models have been instrumental in elucidating the pathogenesis of severe acute respiratory syndrome coronavirus 2 (SARS-CoV-2) infection and in testing coronavirus disease 2019 (COVID-19) vaccines and therapeutics. Wild-type (WT) mice are not susceptible to many SARS-CoV-2 variants, and therefore, transgenic K18-hACE2 mice have emerged as a standard model system. However, this model is characterized by a severe disease, particularly associated with neuroinfection, which leads to early humane endpoint euthanasia. Here, we established a novel knock-in (KI) mouse model by inserting the original K18-hACE2 transgene into the collagen type I alpha chain (COL1A1) locus using a recombinase-mediated cassette exchange (RMCE) system. Once the Col1a1-K18-hACE2 mouse colony was established, animals were challenged with a B.1 SARS-CoV-2 (D614G) isolate and were monitored for up to 14 days. Col1a1-K18-hACE2 mice exhibited an initial weight loss similar to the K18-hACE2 transgenic model but did not develop evident neurologic clinical signs. The majority of Col1a1-K18-hACE2 mice did not reach the pre-established humane endpoint, showing a progressive weight gain 9 days postinfection (dpi). Importantly, despite this apparent milder pathogenicity of the virus in this mouse model compared to the K18-hACE2 transgenic model, high levels of viral RNA were detected in the lungs, oropharyngeal swab, and nasal turbinates. Moreover, the remaining lesions and inflammation in the lungs were still observed 14 dpi. In contrast, although low-level viral RNA could be detected in a minority of Col1a1-K18-hACE2 animals, no brain lesions were observed at any timepoint. Overall, Col1a1-K18-hACE2 mice constitute a new model for investigating SARS-CoV-2 pathogenesis and treatments, with potential implications for studying long-term COVID-19 sequelae.

**IMPORTANCE:**

K18-hACE2 mice express high levels of the human protein angiotensin-converting enzyme 2 (ACE2), the receptor for severe acute respiratory syndrome coronavirus 2 (SARS-CoV-2), and are therefore susceptible to infection by this virus. These animals have been crucial to understanding viral pathogenesis and to testing coronavirus disease 2019 (COVID-19) vaccines and antiviral drugs. However, K18-hACE2 often dies after infection with initial SARS-CoV-2 variants, likely due to a massive brain infection that does not occur in humans. Here, we used a technology known as knock-in (KI) that allows for the targeted insertion of a gene into a mouse, and we have generated a new human ACE2 (hACE2) mouse. We have characterized this new animal model demonstrating that, upon challenge with SARS-CoV-2, the virus replicates in the respiratory tract, damaging lung tissue and causing inflammation. In contrast to K18-hACE2 mice, only limited or no brain infection could be detected in this new model. After 14 days, most animals recovered from the infection, although lung tissue lesions were still observed. This new model could be instrumental for the study of specific disease aspects such as post-COVID-19 condition, sequelae, and susceptibility to reinfection.

## INTRODUCTION

Severe acute respiratory syndrome coronavirus 2 (SARS-CoV-2) is the etiologic agent of the coronavirus disease 2019 (COVID-19). The COVID-19 pandemic fueled an unprecedented collaborative research effort to develop therapeutics and vaccines. Animal models that recapitulate key clinical and pathological features of COVID-19 have played a pivotal role in testing novel vaccines, antivirals, and other treatments (1).

The spike glycoprotein of SARS-CoV-2 uses the angiotensin-converting enzyme 2 (ACE2) as a receptor to enter host cells (2). ACE2 is expressed on the surface of cells of different organs such as the lungs, intestines, kidneys, and heart. However, the spike glycoprotein from ancestral SARS-CoV-2 strains does not efficiently bind to mouse ACE2 (mACE2), rendering wild-type (WT) mice resistant to infection. Therefore, several strategies were used to develop mouse models susceptible to infection. These strategies include viral adaptation (3) or the introduction of the human ACE2 (hACE2) receptor via viral vectors and transgenic approaches (4–8).

Both K18-hACE2 transgenic mice and Golden Syrian hamsters (GSHs) became reference animal models for investigating SARS-CoV-2 pathogenesis *in vivo*. They mainly differ in pathogenesis: GSHs, which are naturally susceptible to SARS-CoV-2 infection, recapitulate a milder disease phenotype (1). The K18-hACE2 mouse model was originally developed for the study of SARS-CoV, which also targets hACE2. It was created by random insertion of multiple copies of the *hACE2* gene under the control of the human cytokeratin 18 gene promoter (*KRT18* or K18). This promoter allows for high-level expression and is specific to epithelial cells, including those in the airways (9, 10). This approach allows the infection of mice by SARS-CoV-2 while maintaining the activity of the mACE2 receptor. The infection of K18-hACE2 mice with pre-Omicron variants of SARS-CoV-2 results in progressive weight loss and severe clinical signs, by 3–5 days postinfection (dpi), and often requires euthanasia by 5–7 dpi. The lethality in this model is dose and SARS-CoV-2-variant dependent (10, 11), and has been associated with neuroinvasion and extensive brain infection (9, 12–14). Neuroinvasion in K18-hACE2 transgenic mice has been linked to the random gene insertion and the high number of inserted gene copies, around eight in the commercial *B6.Cg-Tg(K18-ACE2)2Prlmn/J-K18-hACE2* mouse (10), leading to altered expression levels and tissue distribution of the receptor (15). Specifically, a higher number of gene copies correlate with a worse disease prognosis and encephalitis in this model, following SARS-CoV infection (16). These neurological lesions leading to death in K18-hACE2 mice are not consistent with the pathology of COVID-19 in humans. Although infection of the central nervous system (CNS) can be detected post-mortem in severe COVID-19 cases (17), the presence of SARS-CoV-2 in the CNS does not seem to be associated with the severity of neuropathological alterations in humans (17, 18). Therefore, new mouse models that better recapitulate the human disease, without lethal viral neuroinvasion, could complement other existing animal models (hamster or non-human primates) (1).

Knock-in (KI) models are characterized by the targeted insertion of a defined gene copy number in a specific locus, therefore allowing for a more accurate and predictable expression of the transgene. In this work, we used a recombinase-based approach to insert the original K18-hACE2 transgene into the collagen type I alpha chain (COL1A1) locus to generate a Col1a1-K18-hACE2 KI mouse. To characterize this new model, mice were challenged with SARS-CoV-2 B.1 (D614G) isolate, and pathogenicity was compared with the well-characterized K18-hACE2 transgenic mouse model. After the viral challenge of the Col1a1-K18-hACE2 mice, we confirmed viral replication and histological lesions in the lungs but minimal viral neuroinvasion. This new model could be useful to study specific disease aspects, such as post-COVID-19 condition or sequelae, which are particularly associated with severe infections that occurred during the early phases of the pandemic.

## RESULTS

### Assessment of hACE2 expression in the Col1a1-K18-hACE2 mice

**Fig 1:**
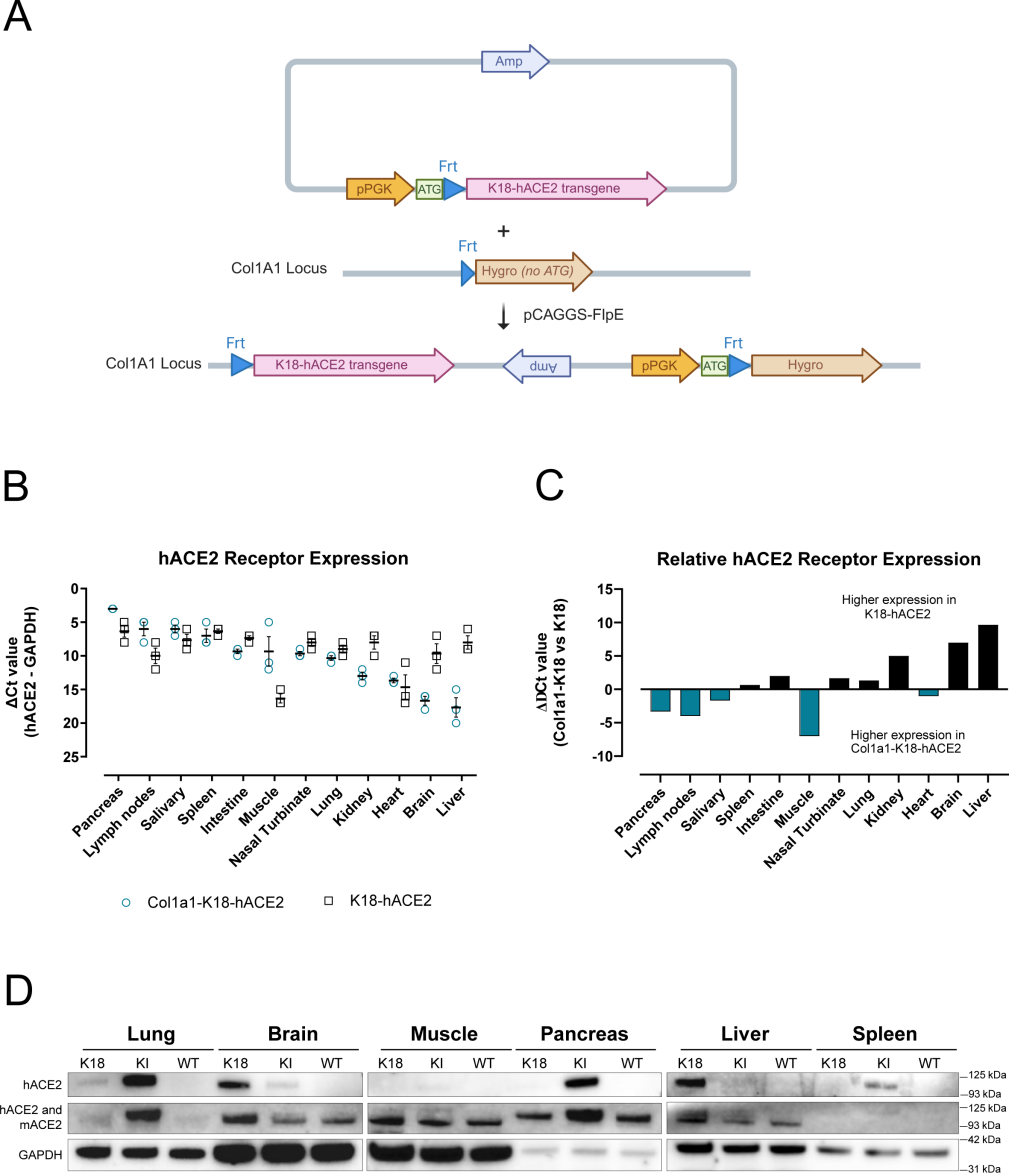
Expression of hACE2 in the Col1a1-K18-hACE2 KI model. (**A**) Schematic representation of the insertion strategy. The original K18-hACE2 transgene was inserted into the collagen COL1A1 locus using a recombinase-mediated cassette exchange (RMCE) FLP-FRT system in KH2 cells via blastocyst injection. pPGK-ATG-frt plasmid: vector backbone with ampicillin-resistant gene (Amp), transcription start site (ATG), and Flippase recognition target (Frt). Hygro: hygromicin resistance gene. pCAGGS-FlpE: expression plasmid for FLPe recombinase expression. (**B**) Relative quantification of hACE2 receptor expression to GAPDH expression in uninfected Col1a1-K18-hACE2 (blue empty dot, *n* = 3) and K18-hACE2 males (black empty square, *n* = 3). Delta Ct values are inversely shown to facilitate interpretation. The lower the absolute number, the higher the relative expression. Solid line and bars represent the mean and SEM. (**C**) Relative comparison of hACE2 receptor expression in Col1a1-K18-hACE2 versus K18-hACE2 mice (*n* = 3 each, males). The higher receptor expression in Col1a1-K18-hACE2 (negative values in *y*-axis) is marked by blue bars, and the higher expression in K18-hACE2 (positive values in *y*-axis) is marked in black bars. (**D**) Western blot analysis of hACE2, mACE2, and GAPDH in representative tissues. hACE2 signal was obtained using a specific monoclonal antibody (top panels); mACE2 and hACE2 signal were obtained with a cross-reactive polyclonal antibody (middle panel), and anti-GAPDH was analyzed as a reference (bottom panels). Molecular weight markers are shown on the right.

We generated a new hACE2 KI mouse model through the addition of the original K18-hACE2 transgene, kindly supplied by Paul McCray (9), at the COL1A1 locus using a recombinase-mediated system (Fig. 1A) (19). Traditional transgenic techniques rely on the random genomic insertion of an unpredictable number of transgene copies. This frequently results in a highly variable level and pattern of transgene expression, due to *cis*-acting regulatory elements at the site of insertion. A single copy of a transgene, inserted into the well-characterized COL1A1 locus, is expected to result in a more predictable level and pattern of expression of the transgene sequence. To confirm the correct expression of the transgene in this KI model, we initially quantified *hACE2* transcripts relative to the housekeeping gene GAPDH by reverse transcription-quantitative PCR (RT-qPCR) in RNA samples extracted from 12 different tissues. The expression of the hACE2 mRNA in K18-hACE2 mice was similar among the most tested tissues except in the muscle and heart, which showed lower values (Fig. 1B). In contrast, hACE2 mRNA expression in Col1a1-K18-hACE2 mice was more heterogenous, with the lowest expression observed in the brain and liver. When comparing the relative expression of hACE2 mRNA between each model, we observed higher expression in the pancreas, lymph nodes, and muscle of Col1a1-K18-hACE2 mice. Conversely, in the kidney, brain, and liver, hACE2 mRNA expression was higher in the K18-hACE2 mouse model. Minor differences were observed in the rest of the tissues analyzed (Fig. 1C). To complement mRNA expression data, protein expression was assessed by Western blot (WB) in six different tissues using a monoclonal antibody specific for hACE2 (20) and a polyclonal antibody recognizing both hACE2 and mACE2. GAPDH was used as a control, and tissues from Col1a1-K18-hACE2, K18-hACE2, and WT mice were analyzed. Data showed undetectable protein expression in the muscle and higher hACE2 expression in the lung, pancreas, and spleen of Col1a1-K18-hACE2 mice compared to K18-hACE2 animals (Fig. 1D). Conversely, as observed by RT-qPCR, a higher hACE2 expression was detected in K18-hACE2 brain and liver compared to Col1a1-K18-hACE2 samples (Fig. 1D).

### Survival of Col1a1-K18-hACE2 mice following SARS-CoV-2 B.1 infection

**Fig 2:**
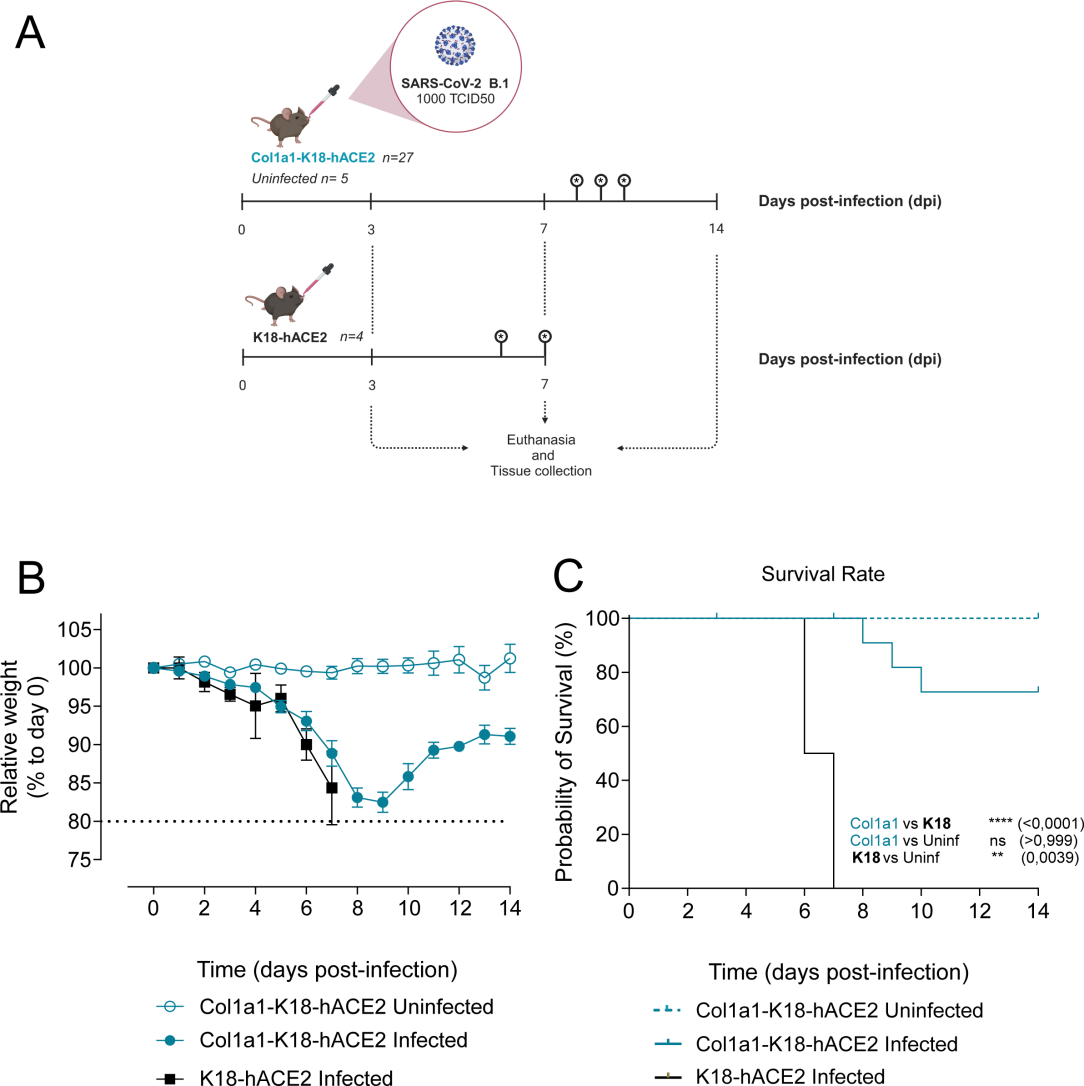
Experimental setting and progression of SARS-CoV-2 infection in Col1a1-K18-hACE2 and K18-hACE2 mouse models. (**A**) Schematic representation of the experimental setting. Knock-in Col1a1-K18-hACE2 (*n* = 27) and transgenic K18-hACE2 mice (*n* = 4) were intranasally challenged with a 1,000 TCID_50_ dose of a B.1 SARS-CoV-2 isolate. A Col1a1-K18-hACE2-uninfected control group (*n* = 5) was challenged with PBS. Mice were monitored for weight loss and clinical signs for 14 dpi. Euthanasia was performed at 3, 7, and 14 dpi or upon fulfilment of humane endpoint criteria, for sample and tissue collection (*n* = 8 per timepoint). Infections were performed in two separate experiments between January and June 2022. Created with Biorender.com. (**B**) Relative body weight follow-up referred to day 0. Col1a1-K18-hACE2 uninfected (blue empty dot), Col1a1-K18-hACE2 infected (blue dot), and K18-hACE2 infected (black square). Solid lines and bars represent the mean ± SD. (**C**) Survival (Kaplan-Meier). All K18-hACE2-infected animals (*n* = 4) had to be euthanized due to endpoint criteria by 7 dpi, and only three were infected with Col1a1-K18-hACE2 at 8, 9, and 10 dpi. No uninfected Col1a1-K18-hACE2 had to be euthanized under this criterion. Col1a1-K18-hACE2 uninfected (blue dashed line), Col1a1-K18-hACE2 infected (blue line), and K18-hACE2 infected (black line). Statistical differences were identified using a log-rank (Mantel-Cox) test (<0.0001), followed by individual comparisons (**P < 0.005, ****P < 0.0001).

Col1a1-K18-hACE2 mice (*n* = 27) and K18-hACE2 mice (*n* = 4) were challenged intranasally with a SARS-CoV-2 B.1 (D614G) isolate. Uninfected KI animals, used as a control, received an intranasal dose of phosphate-buffered saline (PBS) (*n* = 5). Animal weight and clinical signs were monitored daily for 14 days after infection. The endpoints to collect samples were set at 3, 7, and 14 dpi (*n* = 8 Col1a1-K18-hACE2 mice per endpoint) or upon fulfilment of humane endpoint criteria (Fig. 2A).

Consistent with previous studies in transgenic animals with the B.1 SARS-CoV-2 variant, weight loss could be observed in both animal models starting around 3 dpi (Fig. 2B) (13). In K18-hACE2 transgenic animals, the decrease in weight was associated with the progressive appearance of severe clinical signs: decrease in mobility, dyspnea, and neurological effects (Table S1A), fulfilling the humane endpoint criteria and leading to the euthanasia of all K18-hACE animals by 6 or 7 dpi (Fig. 2C). In contrast, while Col1a1-K18-hACE2 mice underwent weight loss, they did not show severe clinical signs. Specifically, we measured an increase in the appearance score and the lack of responsiveness score for the two animal models but no neurological sign for any of the Col1a1-K18-hACE2 mice (Table S1B). Three Col1a1-K18-hACE2 animals had to be euthanized (exitus) exclusively due to their weight loss exceeding the 20% limit (humane endpoint) at 8, 9, and 10 dpi. Convalescent animals started to regain weight after 9 dpi, reaching 90% of their initial weight by 14 dpi (Fig. 2B and C). These results indicate that B.1 SARS-CoV-2 infection is significantly less pathogenic in Col1a1-K18-hACE2 mice than in K18-hACE2 transgenic mice (Fig. 2C).

### SARS-CoV-2 B.1 replicates in the respiratory tract of Col1a1-K18-hACE2 KI mice

**Fig 3:**
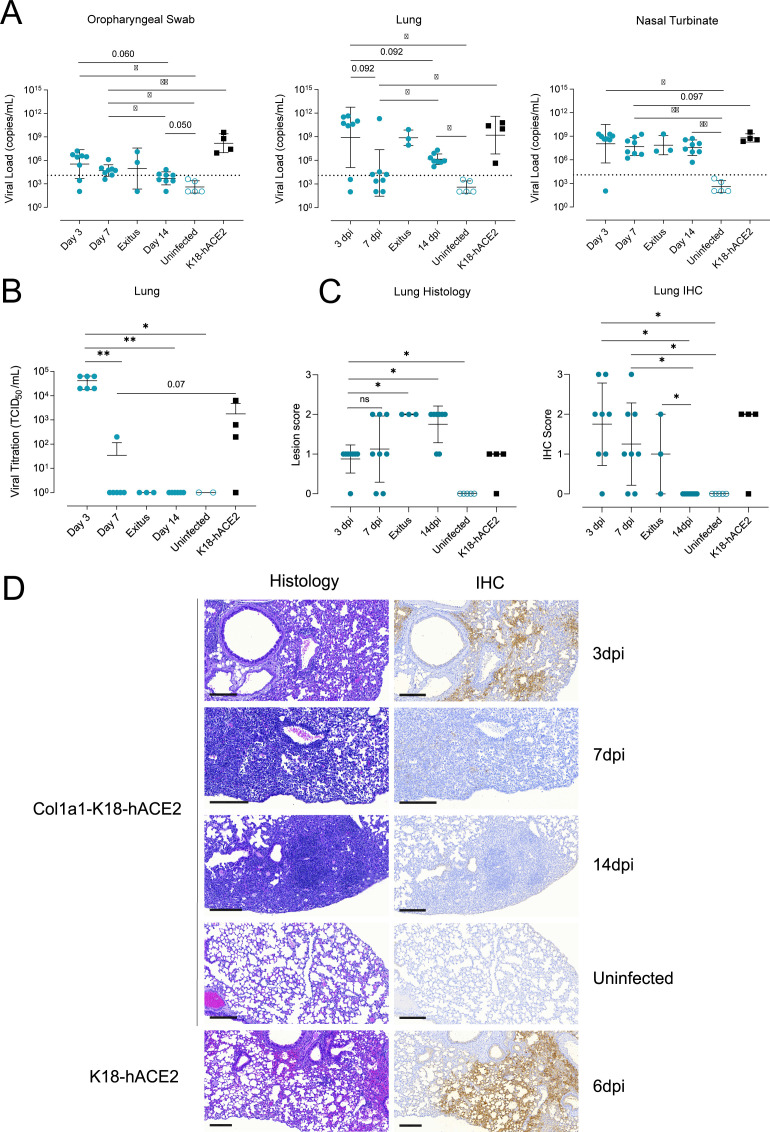
Progression of SARS-CoV-2 infection in Col1a1-K18-hACE2 KI mice. Col1a1-K18-hACE2 (blue circles, *n = 27*) and K18-hACE2+ mice (black squares, *n = 4*) were inoculated with 1,000 TCID_50_ of a SARS-CoV-2 B.1 isolate (full shapes) or uninfected (empty shapes of each color, *n = 5*). Animals were euthanized at 3 dpi (*n* = 8), 7 dpi (*n* = 8), 14 dpi (*n* = 8), or upon fulfilment of humane endpoint criteria (*n* = 3, euthanized at 8, 9, and 10 dpi). (**A**) SARS-CoV-2 viral RNA loads (copies per milliliter) of oropharyngeal swab, lung, and nasal turbinate samples. Dashed lines represent the limit of detection, established by 2SD of uninfected animals. Statistical differences were identified using a Peto-Peto left-censored test (*P < 0.05, **P < 0.005). (**B**) Viral titration of replicative virus (TCID_50_/mL) in lung samples from B.1-infected mice at different endpoints in Vero E6 cells on day 5 of culture. Titers were compared using an independence asymptotic generalized Pearson chi-squared test for ordinal data (*P < 0.05, **P < 0.005). (**C**) Lung histopathological scoring of bronchointerstitial pneumonia (left) and SARS-CoV-2 NP immunohistochemical (IHC) scoring (right) in both models at 3, 7, and 14 dpi and endpoint. Statistical differences were identified using an independence asymptotic generalized Pearson chi-squared test for ordinal data (*P < 0.05). (**D**) Representative lung histology and IHC pictures of both models at 3, 7, and 14 dpi and endpoint. Images show low-power magnification bars (200 µm).

To fully characterize the levels of viral replication in the respiratory tract, samples from oropharyngeal swab, middle right lung lobes, and nasal turbinates (nasoturbinate, maxilloturbinate, and ethnoturbinates) were collected at 3, 7, and 14 dpi or at the humane endpoint (exitus) in all Col1a1-K18-hACE2 mice (*n* = 32). Samples from K18-hACE2 transgenic mice obtained at euthanasia (6 and 7 dpi) were used as reference (*n* = 4). Viral RNA analysis in oropharyngeal swab, middle right lung lobes, and nasal turbinates revealed widespread infection in both K18-hACE2 and Col1a1-K18-hACE2 mice in all mentioned tissues across two independent experiments (Fig. 3A). Viral loads (VLs, defined here as cell-free viral RNA) in the nasal turbinates were similar in both animal models, with some viral RNA still detected at 14 dpi in Col1a1-K18-hACE2 mice. RNA levels in oropharyngeal swabs and lung tissue peaked at 3 dpi in Col1a1-K18-hACE2 mice and tended to decay over time up to 14 dpi. To further confirm viral clearance, titration of replication-competent virus was performed in middle right lung lobe samples. Similar to the VL levels, viral titers peaked at 3 dpi, followed by a quick and significant decrease (Fig. 3B, 3 dpi vs 7 dpi: *P* = 0.00178), concomitant with the elicitation of neutralizing antibodies (Fig. S1). Col1a1-K18-hACE2 animals euthanized at the humane endpoint at 8–10 dpi still displayed a high viral RNA in the lungs, which could explain their more pronounced weight loss and early fulfilment of endpoint criteria. However, we could not recover infectious viruses from these samples (Fig. 3B), suggesting that detected viral RNA represents residual genetic material in cells dying by viral or immune-mediated mechanisms. Consistently, as described in humans (21), high titers of neutralizing antibodies were observed in these animals (Fig. S1).

To characterize histopathological changes, right superior, inferior, and post-caval lung lobes and left lung from both animal models were formalin fixed and analyzed by histology and immunohistochemistry (IHC) to score the presence and extension of lesions and SARS-CoV-2 nucleoprotein antigen (NP), respectively. Histological lesions were semi-quantified as none, mild, moderate, or severe (score 0–3), and the detection of viral antigen as lack, low, moderate, or high (score 0–3). In Col1a1-K18-hACE2 KI mice, histopathological analysis showed the development of bronchointerstitial pneumonia characterized by multifocal increased thickness of interalveolar wall, presence of macrophage-like cells into alveoli surrounding bronchi and bronchiole, and hyperplasia of type II pneumocytes. These lesions evolved from mild and multifocal by 3 dpi to moderate in most of the animals by 14 dpi, also with increasing lymphoplasmacytic infiltration until the end of the study. The extension of hyperplasia of type II pneumocytes was barely detected at 3 dpi, mild by 7 dpi, and moderate by 14 dpi. Therefore, an increase in lung lesion average was observed over time (Fig. 3C and D). Animals reaching the humane endpoint displayed areas of severe bronchointerstitial pneumonia (Fig. S2). IHC detection of SARS-CoV-2 NP in the lungs of Col1a1-K18-hACE2 KI mice was consistent with the VL analyses, showing the highest score early upon infection (3 dpi). Antigen detection decreased with time, showing apparent clearance by 14 dpi (Fig. 3C and D). However, animals reaching the humane endpoint at 8, 9, and 10 dpi showed mild to moderate SARS-CoV-2 antigen staining mainly located in bronchiolar epithelium from different bronchioles (Fig. S2).

**Fig 4:**
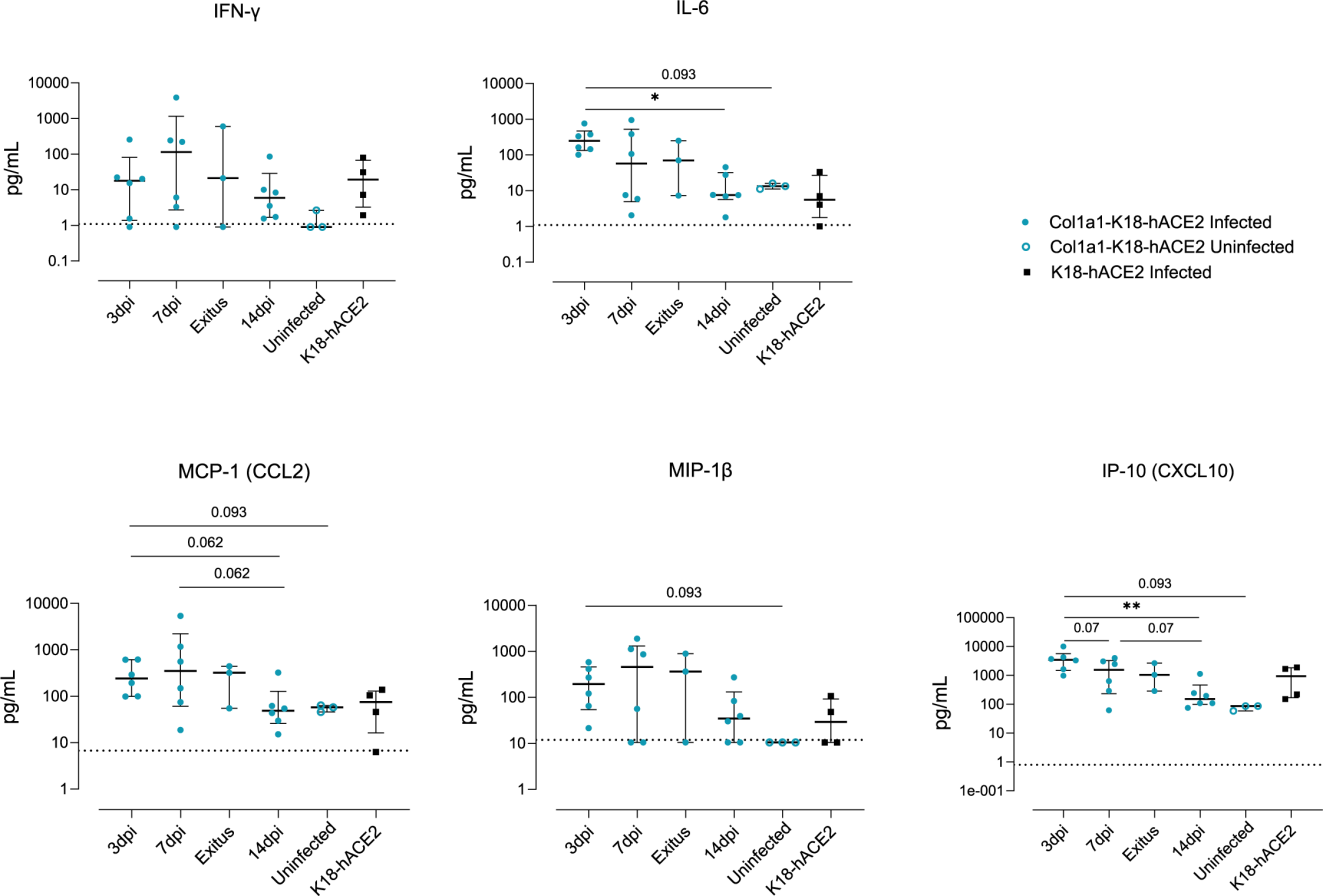
Inflammatory response in the lung. The concentration of inflammatory cytokines in lung extracts in the knock-in and transgenic models at 3, 7, and 14 dpi or endpoint is shown. Col1a1-K18-hACE2-infected (blue full circles, *n = 23*), uninfected (blue empty circles, *n* = 3), and K18-hACE2-infected mice (black full squares, *n = 4*). The bar shows the median with an interquartile range. The limit of detection for each cytokine is indicated by dotted lines. Statistical differences were identified using a Kruskal-Wallis test and a Conover’s nonparametric all-pairs comparison test (*P < 0.05, **P < 0.005).

To assess infection-driven inflammation, we analyzed the levels of IL-6 and IFNγ cytokines, which are involved in inflammation and immune activation, and the chemokines IP-10, MCP-1, and MIP-1β, which are primarily involved in the recruitment of immune cells. Cytokine and chemokine levels were measured by Luminex in middle right lung lobe samples from both animal models at the indicated timepoints. While all markers were increased in infected animals compared to uninfected controls, IP-10 and IL-6 peaked at 3 dpi and significantly decreased by 14 dpi in Col1a1-K18-hACE2 KI mice (Fig. 4). In contrast, IFNγ, MCP-1, and MIP1β peaked at 7 dpi and decayed afterward, although no statistical differences were observed among the analyzed timepoints. Overall, levels of inflammatory mediators at 7 dpi in Col1a1-K18-hACE2 KI mice were comparable to K18-hACE2 transgenic mice at the time of euthanasia (6 and 7 dpi). Residual levels of IFNγ and MIP1β were observed at 14 dpi in some Col1a1-K18-hACE2 mice, probably associated with the remaining tissue lesions at this timepoint, because no viral antigen could be detected (Fig. 3C and D).

### SARS-CoV-2 B.1 replication in other tissues

**Fig 5:**
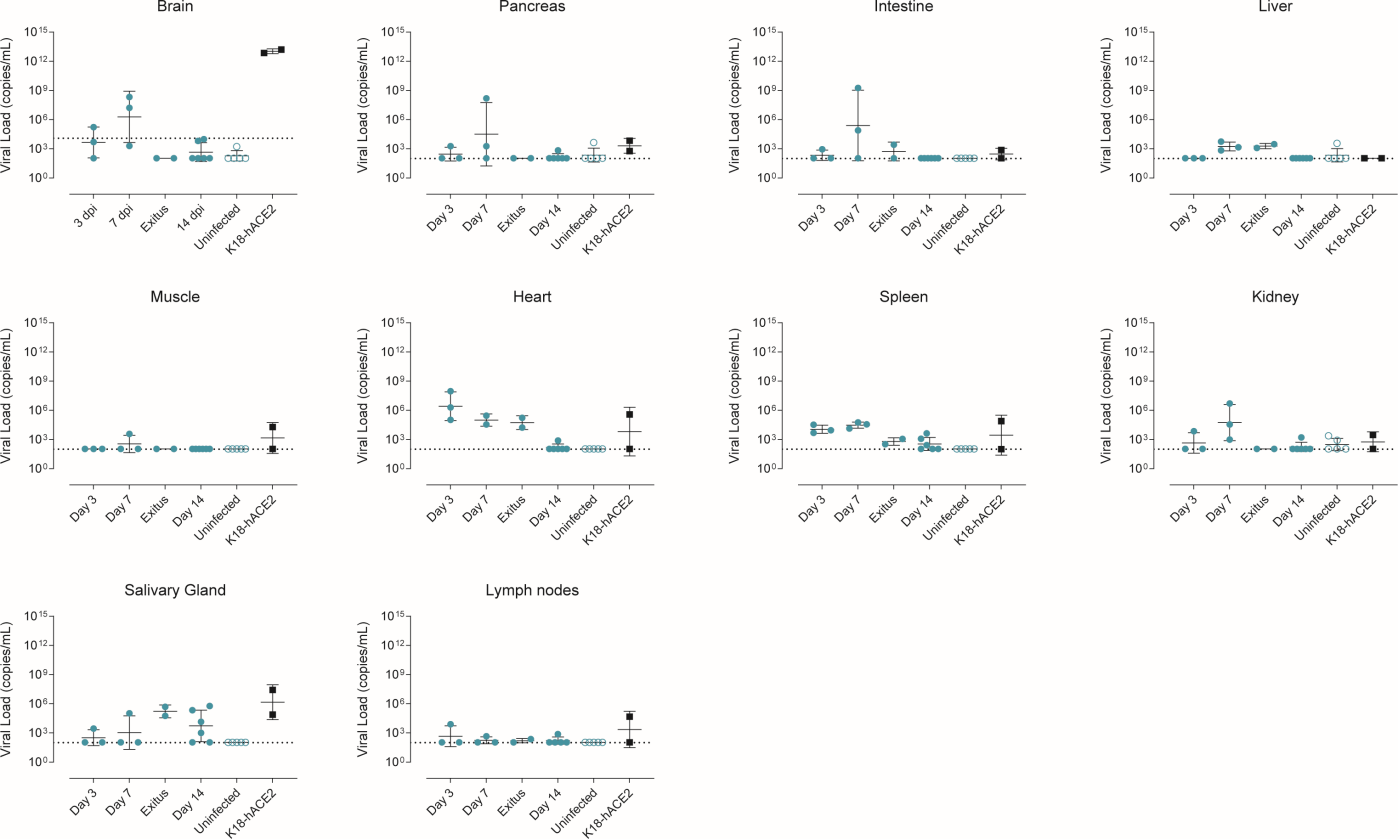
Tissue tropism of SARS-CoV-2 in Col1a1-K18-hACE2 and K18-hACE2 mice. SARS-CoV-2 viral RNA loads (copies per milliliter) were analyzed by RT-qPCR in Col1a1-K18-hACE2 mice tissues collected at 3, 7, and 14 dpi (*n* = 3 per timepoint) or upon fulfilment of humane endpoint criteria (*n* = 1 at 8 dpi, *n* = 1 at 9 dpi). K18-hACE2 tissues were collected at endpoint at 6 (*n* = 1) and 7 (*n* = 1) dpi. Solid lines show the mean ± SD. Dashed lines represent the limit of detection, established by 2SD of uninfected animals.

To explore the extent of viral infection in both animal models, VLs were quantified in a large set of samples (brain, muscle, intestine, liver, kidney, pancreas, salivary glands, lymph nodes, and spleen) collected from a subset of animals (*n* = 20). In both models, VLs were mostly undetected in most tissues except for low levels found in the heart and salivary glands (Fig. 5). The analysis of brain samples showed a high VL exclusively in K18-hACE2 transgenic animals. However, the initial screening of brain tissue from Col1a1-K18-hACE2 mice showed a low but detectable VL in one animal at 3 dpi and two animals at 7 dpi. In sharp contrast, euthanized animals, which showed the highest weight loss and lung VL, lacked detectable VL in the brain (Fig. 5).

### Col1a1-K18-hACE2 KI mice do not develop massive brain infection

**Fig 6:**
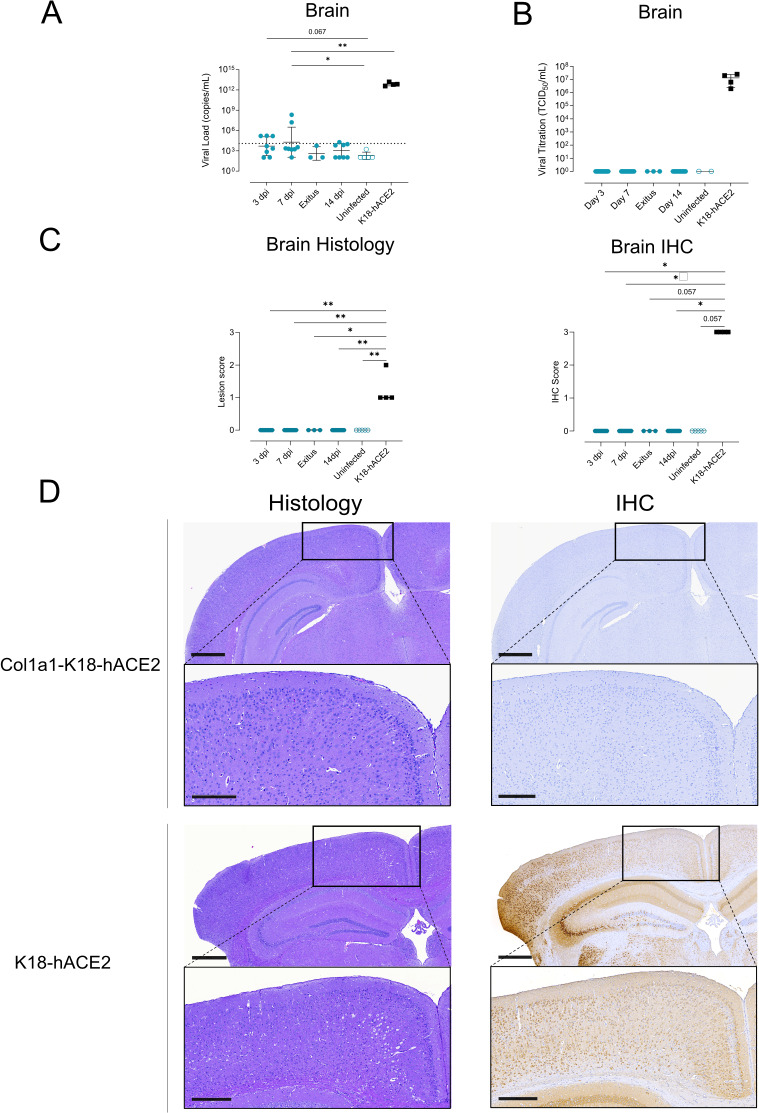
Progression of SARS-CoV-2 infection in the brain of Col1a1-K18-hACE2 KI mice. Col1a1-K18-hACE2 (blue circles, *n = 27*) and K18-hACE2+ mice (black squares, *n = 4*) were inoculated with 1,000 TCID_50_ of a B.1 SARS-CoV-2 isolate (full shapes) or were uninfected (empty shapes, *n* = 5) and were monitored until 14 dpi. Samples were collected at 3, 7, and 14 dpi and at endpoint. (**A**) SARS-CoV-2 viral RNA loads (copies per milliliter) in brain extracts. Dashed lines represent the limit of detection, established by 2SD of uninfected animals. Statistical differences were identified using a Peto-Peto left-censored samples test with correction for multiple comparisons. Solid lines and bars represent the mean and SD. (**B**) Viral titration of replicative virus (TCID_50_/mL) in brain samples of B.1-infected mice at different endpoints in Vero E6 cells on day 5 of culture. Solid lines and bars represent the mean and SD. (**C**) Brain histopathological scoring of multifocal lymphoplasmacytic meningo-encephalitis in the brain (left) and SARS-CoV-2 NP IHC scoring (right) in the brain of both models. Statistical differences were identified using an independence asymptotic generalized Pearson chi-squared test for ordinal data (*P < 0.05, **P < 0.01). (**D**) Representative images of brain histology and IHC images of both hACE2+ mouse models. Images show low-power magnification (top; bars: 600 µm) and medium-power magnification (bottom; bars: 300 µm). Col1a1-K18-hACE2 KI mice samples shown were collected at 7 dpi, while K18-hACE2 samples were collected at humane endpoint (6–7 dpi).

To further investigate the level of viral infection in the brain (Fig. 5), we performed a longitudinal virological and histological analysis of brain samples in all animals (*n* = 32). Considering the full set of data, VL was detected at low levels in 3 of 8 Col1a1-K18-hACE2 mice at 3 dpi, and 2 out of 8 Col1a1-K18-hACE2 KI mice at 7 dpi. No VL was found in the additional animal that reached the humane endpoint. In contrast, all K18-hACE2 brain samples showed a significantly higher VL at euthanasia (6–7 dpi) (Fig. 6A). However, no infective virus could be found in any Col1a1-K18-hACE2 mice at any timepoint by viral titration, while all K18-hACE2 presented high viral titers at 6–7 dpi (Fig. 6B).

For histological analysis, formalin-fixed brain samples were scored in a blinded fashion as described above. Histopathological analysis of the brain in the Col1a1-K18-hACE2 model showed no lesions at any of the analyzed timepoints nor those animals that required humane endpoint (Fig. S2). In contrast, brain samples from K18-hACE2-infected animals showed lesion scores between 1 and 2 (Fig. 6C, left). Furthermore, no detection of SARS-CoV-2 NP antigen in the brain of Col1a1-K18-hACE2 mice was observed by IHC at any timepoint (Fig. 6C) or at the humane endpoint (Fig. S1), while K18-hACE2 mice showed a high density of antigen diffused throughout the tissue (score 3) at euthanasia (Fig. 6C, right). Brain changes in the transgenic model were consistent with the widely reported pathology induced by initial SARS-CoV-2 variants (12, 14, 22) and were observed alongside the detection of viral antigen in the brain (Fig. 6D).

## DISCUSSION

In this study, we characterized a novel hACE2 KI murine model by evaluating the course of infection with the B.1 D614G SARS-CoV-2 variant with a direct comparison to the well-described K18-hACE2 transgenic mouse model, in which this variant is known to be highly pathogenic. The most relevant findings included the susceptibility of the KI model to SARS-CoV-2 infection and the reduced lethality imputed to the reduced viral replication and lesions in the brain.

The development of this KI model began at the start of the COVID-19 pandemic as an alternative to the K18-hACE2 transgenic mouse model. Even though the transgenic model had already been established and used in previous SARS-CoV studies (9), the availability of these animals was limited at first, as colonies needed to be restarted from cryopreserved embryos. In this context, several laboratories developed new hACE2 murine models for SARS-CoV-2 infection. Expression of the hACE2 transgene was achieved through various methods, including directed insertion using CRISPR/Cas9 technology (20) using different promoters such as hepatocyte nuclear factor-3/forkhead homolog 4 (Hfh4) promoter (5) or the mouse ACE2 promoter (6), and by infecting mice with replication-defective adenovirus encoding hACE2 (3). In this work, we followed a different strategy wherein a single copy of the transgene was inserted into the COL1A1 locus but under the K18 promoter as the original transgenic K18-hACE2 model. The selection of this locus was based on its known ability to provide reliable and ubiquitous expression of inserted sequences (19). Additionally, recombinase-mediated cassette exchange (RMCE) allowed the insertion of a single transgene copy, which should better mimic physiological expression, potentially impacting lethality through the reduction of the ectopic expression and the modification of tissue distribution of the hACE2 receptor. A recent study identifying a correlation between lower hACE2 copy number and reduced pathogenicity further supports our initial hypothesis (23).

When compared with K18-hACE2 transgenic mice, B.1 infection of the new Col1a1-K18-hACE2 KI mouse model resulted in much less severe clinical signs with better responsiveness to stimuli, better physical appearance, and no neurological signs. These observations were consistent with a lower viral replication in the brain of Col1a1-K18-hACE2 KI mice, as summarized in Figure S3. Focusing on the respiratory tract, viral replication was observed in both models, although VL and antigen detection were, in general, lower at 7 dpi in Col1a1-K18-hACE2 KI mice. In contrast, lung tissue lesions and inflammation were fully comparable in both models at this timepoint (Fig. S3).

To explain the different pathogenicity between each model, hACE2 expression was analyzed in different tissues. At the mRNA level, the expression in the lungs and nasal turbinates was comparable or higher in Col1a1-K18 hACE2 mice, while the brain and liver showed lower hACE2 expression, as reported for other transgenic approaches (23, 24). The expression of hACE2 protein showed additional differences; it confirmed a lower expression in the brain and liver, and a higher expression in the pancreas of Col1A1-K18-hACE2 animals but revealed a higher expression in the lung and spleen. In general, brain hACE2 expression in this new KI model seems to be closer to the physiological situation in humans, which showed a low expression in the CNS, based on several techniques including transcriptomics and IHC (25). Among the clinical parameters analyzed in Col1a1-K18-hACE2 KI-infected mice, we found that the initial weight loss was similar to that of the K18-hACE mice but was not accompanied by the increased severity of clinical signs that characterize infection in these transgenic mice (neurological signs, lack of responsiveness, and poor general appearance) (13). Consistent with this observation, the infection of Col1a1-K18-hACE2 KI mice led to partial recovery in weight by 14 dpi in most animals. Importantly, the survival rate of Col1a1-K18-hACE2 KI mice was over 70%, with three euthanized animals exclusively due to a weight loss of more than 20%, which was one of the humane endpoints in our study. These data are in line with other hACE2 KI mouse models in which substantial viral replication within the upper and lower respiratory tracts with limited spread to extrapulmonary organs has been described (8, 23, 26).

Among the tissues analyzed in Col1a1-K18-hACE2 KI-infected mice, we found significant replication of SARS CoV-2 in the respiratory tract, mainly in the lungs. Despite viral clearance in Col1a1-K18-hACE2 KI mice, which was confirmed by viral titration and IHC, lung lesions were similar between both models by 7 dpi and remained at 14 dpi in KI animals recovering from the infection. The impact of viral replication on lung inflammation in Col1a1-K18-hACE2 was confirmed and found to be comparable to the inflammation profile reported in K18-hACE2 transgenic mice (22, 27). Infection of Col1a1-K18-hACE2 KI mice induced high levels of IFNγ, IL-6, IP-10, MCP-1, and MIP-1β cytokines. The longitudinal analysis showed an early impact on IL-6 and IP-10 levels (3 dpi), and a clear trend to normalization by 14 dpi, with some residual inflammation remaining. This is consistent with the presence of lesions at this timepoint, and the immune cell infiltration described by 21 dpi in K18-hACE2 transgenic mice surviving infection (22).

The main difference between the Col1a1-K18-hACE2 KI and the K18-hACE2 transgenic models was found in neuroinvasion. Residual levels of viral RNA but no replication-competent virus or viral proteins were detected in the brains of Col1a1-K18-hACE2 KI mice. Furthermore, no detectable brain lesions, such as the multifocal lymphoplasmacytic meningoencephalitis reported in K18-hACE2 transgenic mice (14), were detected by histology, and no SARS-CoV-2 nucleoprotein could be evidenced by IHC. Importantly, the three animals that reached humane endpoints did not present any evidence of brain viral replication but showed enhanced lung infection. Considering that the expression of hACE2 is lower in the brain and higher in the lung in Col1a1-K18-hACE2 compared to K18-hACE2, it seems that the targeted insertion of the hACE2 transgene can be a crucial factor leading to lower levels of brain infection and neurological clinical signs, as well as enhanced respiratory pathology in this new model. However, different studies have shown that neuroinvasion is only partially dependent on the expression of hACE2 receptor (12), and that both delivery route and viral dose can play a role in the magnitude of the neuroinvasion (24, 28). In our case, to exclusively analyze the effect of receptor expression, we kept the intranasal administration route and TCID_50_ dose established and characterized in our own and others’ studies to ensure comparability of the results (13, 29).

Our study is a preliminary characterization of a new animal model and has consequently several limitations. The first limitation was the age of the animals used. The Col1a1-K18-hACE2 mice ranged in age from 5 to 11 months, depending on availability at the time of the study. However, although the pathogenicity of SARS-CoV-2 infection has the potential to be higher in older animals, we could not detect significant age differences between euthanized and convalescent animals (mean ± SD age of 9.1 ± 2.0 and 8.0 ± 2.1 months, respectively). Second, the expression of hACE2 has been analyzed at both the mRNA and protein levels in bulk tissues, but further characterization in specific cell types will be necessary to link the expression levels with pathology. Moreover, we have focused on the characterization of one viral variant and a single dose. The B.1 variant was selected for its demonstrated severity in K18-hACE2 mice and its inability to use mACE2 as an entry receptor, unlike B.1.351/Beta and Omicron BA1.1 variants (13, 15). While ancestral and earlier variants (B.1.1.7/Alpha, B.1.351/Beta, B.1.617.2/Delta) were highly pathogenic in K18-hACE2 mice, Omicron BA.1.1 caused a milder infection with no weight loss nor neuroinvasion, therefore reducing lethality in this model (13). However, more recent Omicron subvariants such as BQ.1.1, BA.5, and XBB.1.5 appear to be regaining pathogenicity in K18-hACE2 mice (30–32). Increased lung infection, pro-inflammatory cytokines, and lung pathology are observed with these variants, with data on neuroinvasion and neurovirulence on the latter (32). Similarly, a lower lethality of Omicron variants has been described in KI mice (26, 33). Nevertheless, for the B.1 SARS-CoV-2 variant, the Col1a1-K18-hACE2 model shows similarity with GSH in viral dynamics and pathology, with minimal neuroinvasion. This new model, however, has the advantage of a wider reagent availability, scarce in GSH (1), and easier animal facility allowance.

In summary, several mouse models for the evaluation of antivirals and vaccines have been developed to date, with disease phenotypes ranging from mild to severe COVID-19-like condition. None of these models, however, can fully recapitulate all aspects of the disease as it occurs in humans. The development and further characterization of new animal models, like the Col1a1-K18-hACE2 model, may overcome some of these limitations and provide valuable tools to study certain aspects of COVID-19. Survival of Col1a1-K18-hACE2 KI mice after infection with a highly pathogenic variant could provide a model that better mimics human disease progression and thus could be instrumental for the study of specific disease aspects such as the consequences of inflammation (including loss of taste or smell) of post-COVID conditions (PCC) or sequelae, long-term effects of drug therapies, and susceptibility to reinfection.

## MATERIALS AND METHODS

### Creation of the knock-in model

A new KI mouse model, B6;129S-Col1a1^tm1(K18-hACE2)Irb^/Irsi (Col1a1-K18-hACE2), was created by inserting the original K18-hACE2 transgene into the collagen COL1A1 locus using an RMCE FLP-FRT system in KH2 cells (19). The actual insertion site lies approximately 0.3 kb downstream of the 3′UTR end of COL1A1. As such, the inserted transgene remains identical to the original designed one (9), and the hACE2 cDNA is under the control of the K18 promoter rather than COL1A1. Specifically, the plasmid PGK-ATG-Frt was digested with EcoRV, and the sequence ATCAGACGTCGCTAGCGGCGCGCCGGTACTAGT was inserted to create a multi-cloning site.

The plasmid containing hACE2 under the control of the K18 promoter (K18-hACE2), supplied by Paul McCray, was digested with HpaI and XbaI enzymes. The resulting transgene fragment was isolated, purified, and then cloned into the EcoRV-NheI sites of the modified PGK-ATG-Frt plasmid to generate the targeting vector. The targeting vector was then co-transfected with an Flp expression construct into KH2 cells by electroporation. The cells were then placed under hygromycin selection for 9 days, after which drug-resistant colonies were picked, expanded, and screened for the presence of the targeted transgene. Once confirmed, the modified embryonic stem cells were injected into mouse blastocysts. These injected mouse blastocysts were then placed into recipient foster females via embryo transfer techniques.

### Biosafety approval and virus isolation

The execution of SARS-CoV-2 experiments was approved by the biologic biosafety committee of Germans Trias i Pujol Research Institute (IGTP) and was performed at the Biosafety Level 3 laboratory (BSL-3) of the Comparative Medicine and Bioimage Centre (CSB-20-015-M8; CMCiB, Badalona, Spain).

The B.1 SARS-CoV-2 isolate used in this study was isolated from nasopharyngeal swabs of hospitalized patients in Spain as described elsewhere (34, 35). Briefly, viruses were propagated in Vero E6 cells (CRL-1586; ATCC, Virginia, USA) for two passages and were recovered by supernatant collection. The sequence of the SARS-CoV-2 variant tested is deposited at the GISAID Repository with accession IDs EPI_ISL_510689. EPI_ISL_510689 was the first SARS-CoV-2 virus isolated in Catalonia in March 2020, and compared to the Wuhan/Hu-1/2019 (WH1) strain, this isolate had the S protein mutations D614G, which is associated with the B.1 lineage, and R682L. Viral stocks were titrated on Vero E6 cells to use equivalent TCID_50_/mL using the Reed-Muench method and sequential 1/10 dilutions of the viral stocks as described previously (35).

### Animal procedures and study design

All experiments and sample processing were performed inside the BSL-3 facility. Col1a1-K18-hACE2 hemizygous KI mice were produced and bred at Parc Científic de Barcelona (PCB) by pairing hemizygous males for Tg(K18-ACE2)2Prlmn (or K18-hACE2) with non-carrier C57Bl6/J females. Animals used as control, B6.Cg-Tg(K18-ACE2)2Prlmn/J (or K18-hACE2) hemizygous transgenic mice (034860, Jackson Immunoresearch, West Grove, PA, USA) were bred at CMCiB in the specific pathogen-free (SPF) area by pairing hemizygous K18-hACE2 males with non-carrier C57Bl6/J females. The genotype of the offspring regarding both K18-hACE2 and Col1a1-K18-hACE2 mice was determined by qPCR at the IGTP’s Genomics Platform from tail samples. Both animal models were kept in the BSL-3 facility during the whole experiment, including the acclimatization period. The housing conditions in the BSL-3 room were maintained as follows: a temperature of 22±2°C, humidity levels between 30% and 70%, 20 ACH, a 12 hour dark/light cycle, and access to food and water *ad libitum*.

A total of 36 adult mice aged 5–11 months were used in this experiment, consisting of 32 KI Col1a1-K18-hACE2 mice and 4 transgenic K18-hACE2 mice. All groups were sex balanced. The infections were performed in two separate experiments between January and June 2022. Mice were anesthetized with isoflurane (FDG9623; Baxter, Deerfield, IL, USA) and challenged with a B.1 isolate (27 Col1a1-K18-hACE2 and 4 K18-hACE2 mice). The uninfected control group received PBS (5 Col1a1-K18-hACE2).

Infection was performed using 1,000 TCID_50_ of B.1 SARS-CoV-2 isolate in 50 µL of PBS (25 µL/nostril), or PBS only (25 µL/nostril) for the control group. All mice fully recovered from the challenge and anesthesia procedures. Following the challenge, body weight and clinical signs were monitored daily. Eight animals per group were euthanized at days 3, 7, and 14 dpi or upon fulfilment of human endpoint for viral RNA quantification and histological analyses (Table S2). The endpoint criteria for animal welfare were established based on a body weight loss of more than 20% of the initial body weight and/or the display of moderate-to-severe clinical signs (including neurological signs) in accordance with previous studies (12–14). The evaluated clinical signs included respiration, physical appearance, lack of responsiveness, and neurological signs, which were scored from 0 to 2 depending on the severity (Table S1). Euthanasia was performed under deep isoflurane anesthesia by whole blood extraction via cardiac puncture and cervical dislocation. Oropharyngeal swab, lung, brain, and nasal turbinates were collected for viral and hACE2 RNA quantification, histological, and IHC analyses. For the latter techniques, tissues were fixed by immersion in 10% buffered formalin. An additional set of nine tissues was collected to further characterize the KI model for both viral RNA quantification and hACE2 receptor expression. These tissues included muscle, intestine, liver, kidney, lymph nodes, spleen, heart, pancreas, and salivary glands.

### Tissue sampling and processing

The collected tissues were processed as described by Tarrés-Freixas et al. (13). Briefly, approximately 100 mg of each tissue was collected in 1.5 mL Sarstedt tubes (72607; Sarstedt, Nümbrecth, Germany) containing 500 µL of Dulbecco's modified Eagle's medium (DMEM) (11995065; Thermo Fisher Scientific) supplemented with 1% penicillin–streptomycin (10378016; Thermo Fisher Scientific). A 1.5 mm Tungsten bead (69997; QIAGEN, Hilden, Germany) was added to each tube, and samples were homogenized twice at 25 Hz for 30 seconds using a TissueLyser II (85300; QIAGEN) before being centrifuged for 2 minutes at 2,000 × *g*. Supernatants were then stored at −80°C until the analysis of hACE2 expression and VL by RT-qPCR.

### hACE2 mRNA expression

RNA tissue extraction was performed by using the Viral RNA/Pathogen Nucleic Acid Isolation kit (A42352; Thermo Fisher Scientific), optimized for use with a KingFisher instrument (5400610; Thermo Fisher Scientific), following the manufacturer’s instructions. The expression of the hACE2 receptor in tissue was detected by RT-qPCR with a predefined TaqMan assay with an ACE2 target (Hs01085333_m1; Thermo Fisher Scientific). Mouse *gapdh* gene expression was measured in duplicate for each sample using TaqMan gene expression assay (Mm99999915_g1; Thermo Fisher Scientific) as amplification control. Data were graphed both as the delta-Ct values of hACE2 minus GAPDH for each tissue and the relative expression of the receptor (delta-delta Ct value) of Col1a1-K18-hACE2 minus K18-hACE2 tissues.

### ACE2 protein expression

ACE2 protein expression was assessed by WB. Tissue samples were obtained from uninfected Col1a1-K18-hACE2, K18-hACE2, and WT mice and were flash frozen. Frozen sections of each tissue were sliced by cryostat processing (Leica CM1950) and were homogenized in 100 uL of RIPA buffer for 15 minutes on ice, followed by centrifugation at 1,000 × *g* for 10 minutes. Supernatant was then isolated, and total protein concentration was quantified by Pierce BCA Protein Assay (23225; Thermo Fisher Scientific) following the manufacturer’s instructions.

For WB, 3 µg of lysate total protein (as quantified by Pierce BCA Protein Assay [23225; Thermo Fisher Scientific]) was run in NuPAGE Bis-Tris 4%–12% acrylamide gels (NP0321; Thermo Fisher Scientific). BlueStar pre-stained protein ladder was used as a molecular weight marker (523005; NIPPON Genetics, Tokyo, Japan). Proteins were blotted onto polyvinylidene fluoride (PVDF) membrane (Ref. 1704156; Bio-Rad) using the Trans-Blot Turbo Transfer System (Ref. 1704150; Bio-Rad), and membranes were blocked with EveryBlot Blocking Buffer (12010020; Bio-Rad). Primary antibodies used were anti-hACE2 specific monoclonal (1:1,000; AB108209; Abcam) (20), anti-ACE2 polyclonal (1:1,000; AF933; R&D Systems), and anti-GAPDH (1:5,000; Ab9485; Abcam). Secondary antibodies were donkey anti-rabbit (1:10,000; Ref. 711-036-152; Jackson Immunoresearch) or a donkey anti-goat (1:10,000; Ref. 705-035-147; Jackson Immunoresearch). Membranes were developed with SuperSignal West Femto Maximum Sensitivity Substrate (Ref. 34094; Thermo Fisher Scientific) according to the manufacturer’s instructions and were acquired with ChemiDOC MP Imaging System (12003154; Bio-Rad). Images were processed and merged with the ImageLab 6.0.1 software (Bio-Rad).

### SARS-CoV-2 PCR detection and viral load quantification

Cell-free viral RNA was quantified using RT-qPCR in two sets of tissues: the standard set, which included oropharyngeal swabs, lung tissue, brain tissue, and nasal turbinates; and the extended set, which comprised the muscle (gastrocnemius), intestine (duodenum), liver (median lobe), kidney, pancreas, salivary glands, lymph nodes, and spleen. For RT-qPCR analysis, specific tissue parts were selected while the remainder of each organ was preserved for IHC. For the lung, the middle right lobe was used. Brain tissue included the right frontal part of the cerebrum, extending from the olfactory bulb to the primary motor and somatosensory areas of the cerebral cortex. Nasal turbinates were collected from the right half of the cranium and included nasoturbinate, maxilloturbinate, and ethnoturbinate. All RNA extraction was performed by using the Viral RNA/Pathogen Nucleic Acid Isolation kit (A42352; Thermo Fisher Scientific), optimized for use with a KingFisher instrument (5400610; Thermo Fisher Scientific), following the manufacturer’s instructions. PCR amplification was based on the 2019-Novel Coronavirus Real-Time RT-PCR Diagnostic Panel guidelines and protocol developed by the American Center for Disease Control and Prevention (CDC-006-00019, v.07). Briefly, a 20 µL PCR was set up containing 5 µL of RNA, 1.5 µL of N2 primers and probe (2019-nCov CDC EUA Kit; catalog number 10006770; Integrated DNA Technologies, Coralville, IA, USA), and 10 µL of GoTaq 1-Step RT-qPCR (Promega, Madison, WI, USA). Thermal cycling was performed at 50°C for 15 minutes for reverse transcription, followed by 95°C for 2 minutes, and then 45 cycles of 95°C for 10 seconds, 56°C for 15 seconds, and 72°C for 30 seconds in the Applied Biosystems 7,500 or QuantStudio5 Real-Time PCR instruments (Thermo Fisher Scientific). For absolute quantification, a standard curve was built using 1/5 serial dilutions of a SARS-CoV-2 plasmid (2019-nCoV_N_Positive Control; catalog number 10006625, 200 copies/μL, Integrated DNA Technologies), which was run in parallel with all PCR determinations. VL from each sample was quantified in triplicate, and the mean viral RNA concentration (in copies per milliliter of tissue lysate) was extrapolated from the standard curve and corrected by the corresponding dilution factor. Mouse *gapdh* gene expression was measured in duplicate for each sample using TaqMan gene expression assay (Mm99999915_g1; Thermo Fisher Scientific) as amplification control.

### Viral titration

Lung tissues (middle right lobe) were evaluated for the presence of replicative virus by titration in Vero E6 cells as previously described (14, 35, 36). Briefly, after tissue homogenization, each sample underwent sequential 10-fold dilutions in duplicate, transferred onto a monolayer of Vero E6 cell in a 96-well plate, and incubated at 37°C and 5% CO_2_. Plates were monitored daily under a microscope, and at 5 dpi, wells were evaluated for the presence of cytopathic effects. The amount of infectious virus was calculated by determining the TCID_50_ using the Reed–Muench method.

### Histological and immunohistochemical analyses

Tissue samples were recovered at the designated endpoint (3, 7, and 14 dpi or humane endpoint), representative parts processed for VL, and the rest fixed by immersion in 10% buffered formalin. Lung (right superior, inferior, and post-caval lobes and left lung), nasal turbinates, and brain samples were routinely processed for histological examination, with hematoxylin and eosin-stained slides examined under an optical microscope in a blinded fashion. Brain tissue was cut into two coronal sections, including cerebellum/pons and hemispheres at thalamus level. A semi-quantitative approach based on the amount of inflammation (none, mild, moderate, or severe) was used to assess the damage caused by SARS-CoV-2 infection in mice, following a previously published scoring system (14, 37). Additionally, an IHC technique was employed to detect SARS-CoV-2 NP antigen in nasal turbinates, lung, and brain sections from all animals, using a rabbit monoclonal antibody (40143-R019; Sino Biological, Beijing, China) at a 1:15,000 dilution. The amount of viral antigen in tissues was semi-quantitatively scored in a blinded fashion (low, moderate, and high amount, or lack of antigen detection) (14, 37).

### Cytokine quantification

To assess the viral-driven inflammation in the lung of both animal models, the levels of IP-10, IL-6, IFNγ, MCP-1, and MIP-1β cytokines were analyzed by Luminex in tissue extracts. Lung middle right lobe samples were processed as described above and stored at −80°C until analysis. In Col1a1-K18-hACE2 mice, cytokines were analyzed at 3, 7, and 14 dpi and those euthanized by humane endpoint criteria (exitus). Uninfected Col1a1-K18-hACE2 and K18-hACE2-infected animals were used as reference groups.

Cytokines were measured by Luminex xMAP technology and were analyzed with xPONENT 3.1 software (Luminex Corporation) using the MCYTOMAG-70 kit, according to the manufacturer’s protocol with minor modifications. Briefly, after cytokine staining, samples underwent an overnight incubation on a rocking shaker at 4°C, using 2% paraformaldehyde (PFA) to ensure complete inactivation of any remaining SARS-CoV-2 particles; a fixation that does not alter cytokine quantification (38). Before plate acquisition, the PFA was washed away and replaced with sheath fluid.

### Pseudovirus generation and neutralization assay

To assess the generation of neutralizing antibodies upon SARS-CoV-2 B.1 infection, we used a previously described pseudovirus-based neutralization assay (39). Briefly, in Nunc 96-well cell culture plates (Thermo Fisher Scientific), 200 TCID_50_ of a luciferase-reporter HIV-based pseudovirus bearing the BA.1 SARS-CoV-2 spike was preincubated with fourfold serial dilutions (1/60–1/61,440) of the heat-inactivated (56°C for 30 minutes) serum samples for 1 hour at 37°C. Then, 1 × 10^4^ HEK293T/hACE2 cells treated with DEAE-dextran (Sigma-Aldrich) were added. Results were read after 48 hours using the EnSight Multimode Plate Reader and BriteLite Plus Luciferase reagent (PerkinElmer, USA). The values were normalized, and the ID50 (reciprocal dilution inhibiting 50% of the infection) was calculated as described (40).

### Statistical analyses

All figures were generated using GraphPad Prism 9.0.0. Statistical analyses were performed using R v4.3. Survival rates were estimated with Kaplan-Meier curves and were compared with the log-rank test. Data sets with an abundance of data below the limit of detection, like VL, were analyzed using the Peto-Peto left-censored samples test with correction for multiple comparisons. Histopathological, IHC scores, and viral titrations were compared using an independence asymptotic generalized Pearson chi-squared test for ordinal data. Cytokine titers were compared by a Kruskal-Wallis test, with pairwise comparisons conducted using Conover’s non-parametric test.

## Data Availability

The data supporting the findings of this study are documented within the paper and are available from the corresponding authors upon request.

## SUPPLEMENTAL MATERIAL

Supplemental Material mbio.00720-25-s0001.pdf Figure S1 to S3; Tables S1 and S2.

ASM does not own the copyrights to Supplemental Material that may be linked to, or accessed through, an article. The authors have granted ASM a non-exclusive, world-wide license to publish the Supplemental Material files. Please contact the corresponding author directly for reuse.
